# Use of Wastewater Metrics to Track COVID-19 in the US

**DOI:** 10.1001/jamanetworkopen.2023.25591

**Published:** 2023-07-26

**Authors:** Meri R. J. Varkila, Maria E. Montez-Rath, Joshua A. Salomon, Xue Yu, Geoffrey A. Block, Douglas K. Owens, Glenn M. Chertow, Julie Parsonnet, Shuchi Anand

**Affiliations:** 1 Division of Infectious Diseases and Geographic Medicine, Department of Medicine, Stanford University, Palo Alto, California; 2 Division of Nephrology, Department of Medicine, Stanford University, Palo Alto, California; 3 Department of Health Policy, Stanford University, Stanford, California; 4 US Renal Care, Plano, Texas; 5 Department of Epidemiology and Population Health, Stanford University, Palo Alto, California

## Abstract

**Importance:**

Widespread use of at-home COVID-19 tests hampers determination of community COVID-19 incidence.

**Objective:**

To examine the association of county-level wastewater metrics with high case and hospitalization rates nationwide both before and after widespread use of at-home tests.

**Design, Setting, and Participants:**

This observational cohort study with a time series analysis was conducted from January to September 2022 in 268 US counties in 22 states participating in the US Centers for Disease Control and Prevention’s National Wastewater Surveillance System. Participants included the populations of those US counties.

**Exposures:**

County level of circulating SARS-CoV-2 as determined by metrics based on viral wastewater concentration relative to the county maximum (ie, wastewater percentile) and 15-day percentage change in SARS-CoV-2 (ie, percentage change).

**Main Outcomes and Measures:**

High county incidence of COVID-19 as evidenced by dichotomized reported cases (current cases ≥200 per 100 000 population) and hospitalization (≥10 per 100 000 population lagged by 2 weeks) rates, stratified by calendar quarter.

**Results:**

In the first quarter of 2022, use of the wastewater percentile detected high reported case (area under the curve [AUC], 0.95; 95% CI, 0.94-0.96) and hospitalization (AUC, 0.86; 95% CI, 0.84-0.88) rates. The percentage change metric performed poorly, with AUCs ranging from 0.51 (95% CI, 0.50-0.53) to 0.57 (95% CI, 0.55-0.59) for reported new cases, and from 0.50 (95% CI, 0.48-0.52) to 0.55 (95% CI, 0.53-0.57) for hospitalizations across the first 3 quarters of 2022. The Youden index for detecting high case rates was wastewater percentile of 51% (sensitivity, 0.82; 95% CI, 0.80-0.84; specificity, 0.93; 95% CI, 0.92-0.95). A model inclusive of both metrics performed no better than using wastewater percentile alone. The performance of wastewater percentile declined over time for cases in the second quarter (AUC, 0.84; 95% CI, 0.82-0.86) and third quarter (AUC, 0.72; 95% CI, 0.70-0.75) of 2022.

**Conclusions and Relevance:**

In this study, nationwide, county wastewater levels relative to the county maximum were associated with high COVID-19 case and hospitalization rates in the first quarter of 2022, but there was increasing dissociation between wastewater and clinical metrics in subsequent quarters, which may reflect increasing underreporting of cases, reduced testing, and possibly lower virulence of infection due to vaccines and treatments. This study offers a strategy to operationalize county wastewater percentile to improve the accurate assessment of community SARS-CoV-2 infection prevalence when reliability of conventional surveillance data is declining.

## Key Points

**Question:**

Are wastewater surveillance metrics reported to the Centers for Disease Control and Prevention’s National Wastewater Surveillance System associated with high community case and hospitalization rates of COVID-19 across US counties?

**Findings:**

In this cohort study with a time series analysis of 268 counties in 22 states from January to September 2022, SARS-CoV-2 wastewater metrics accurately reflected high clinical rates of disease early in 2022, but this association declined over time as home testing and vaccination increased.

**Meaning:**

These findings suggest that wastewater surveillance can provide an accurate assessment of county SARS-CoV-2 incidence and may be the best metric for monitoring amount of circulating virus as home testing increases and disease acuity decreases because of vaccination and treatment.

## Teaser

This cohort study uses time series analysis to examine the association of county-level wastewater metrics with COVID-19 case and hospitalization rates nationwide both before and after widespread use of at-home tests.

## Introduction

Rapid determination of COVID-19 incidence within communities can guide screening at hospitals, residential facilities, schools, or communal gatherings; mobilize treatment supplies; and preserve hospital capacity. Public health agencies including the US Centers for Disease Control and Prevention (CDC) relied chiefly on rates of reported new COVID-19 cases and/or hospitalizations to estimate county levels of COVID-19.^1^ As home testing becomes widespread, however, case counts are likely to substantially underestimate disease incidence, to a degree depending on at-home test availability, acceptance, and cost, as well as the severity of disease seen with circulating strains of SARS-CoV-2.^2^ Similarly, since the introductions of vaccines and medications that reduce COVID-19 severity,^3^ tracking hospitalization rates may produce unreliable estimates of disease incidence. The lack of accurate data regarding community infection prevalence leaves high-risk patients at particular risk.

Wastewater surveillance offers a potential solution to the problem of accurate SARS-CoV-2 surveillance because it is agnostic to symptomatic, diagnosed, or reported disease. High-resolution sequencing of wastewater can also identify emerging variants of concern^4^ and estimate the effective reproductive number,^5^ a key predictor of future transmission. For these reasons, many jurisdictions are investing in expanding wastewater surveillance, with over 70 countries and more than 3500 sites reporting data to a central dashboard.^6^ Yet adoption of wastewater surveillance to inform public policy has not yet become widespread, in part because of challenges in interpreting results with shifting detection methods, virus strains, populations served, and wastewater dynamics.^7^ To date, available data evaluating wastewater metrics against cases or hospitalizations in the US are geographically limited, evaluating a single sewershed^8,9^or a few sewersheds grouped regionally.^10^

The CDC’s National Wastewater Surveillance System (NWSS) collates data from a majority of currently operating wastewater testing sites in the US.^11^ The NWSS normalizes wastewater samples to wastewater flow and population size served and relies on viral gene copies per individual in the sewershed as the foundational data unit. This normalization addresses concerns about changes due to weather and differences in sewershed size. Despite normalization and generation of aggregate measures (eg, percentage change in normalized virus concentration in the last 15 days), no interpretation algorithm is provided to inform screening policy. Indeed, the NWSS specifically recommends that “point estimates of community infection based on wastewater measurements should not be used”^12^ to shape policy, largely because the amount of virus shed by individuals with infection into the sewage system has not been well characterized.^13^ Yet, with decreased institutional testing, lower disease virulence for a majority of the immunocompetent disease population, and, as of May 2023, CDC’s discontinuation of publicly shared case metrics, wastewater may be the best (and possibly only) way to understand the dynamics of circulating SARS-CoV-2 virus in communities.

Using data from the NWSS, we sought to evaluate how well national data on wastewater SARS-CoV-2 measures paralleled reported new COVID-19 cases and hospitalizations over time in the US. We hypothesized that the association of wastewater with COVID-19 disease metrics would be greater before widespread home COVID-19 testing and would decrease over time—that is, there would be attenuation of the association between viral transmission and formally reported new case and hospitalization rates. We aimed to determine whether selected wastewater surveillance metrics can be operationalized for future integration with other disease or socioeconomic vulnerability metrics to inform policy decisions regarding resource allocation to areas with high disease prevalence.

## Methods

### Data Sources

#### Wastewater Metrics

In this cohort study with a time series analysis, we obtained publicly available data from the NWSS spanning the Omicron variant dominant period of January to September 2022.^11^ Because this work solely relies on publicly available data that do not carry any protected health information and we did not have access to codes or linkage that could enable individual identification, it is exempt from ethics review and the need for informed consent, as per Common Rule 45 CFR46.102. This study follows the Strengthening the Reporting of Observational Studies in Epidemiology (STROBE) reporting guidelines.

The NWSS reports data on wastewater from public health department–monitored sewersheds serving at least 3000 people. Excluded are sewersheds missing population estimates or that represent single institutions (eg, a university). The sewersheds quantify SARS-CoV2 in unconcentrated wastewater or sludge using either reverse-transcription quantitative polymerase chain reaction (672 sites) or reverse-transcription digital polymerase chain reaction (545 sites); irrespective of sample type and method, sites report virus concentrations per volume.^12^

Publicly reported data include sewershed location identifiers and population served. In an effort to facilitate comparisons, NWSS-aggregated metrics include facility SARS-CoV-2 percentile (ie, an ordered rank of the current virus concentration relative to historic peak and nadir at that facility, hereafter referred to as *wastewater percentile*), percentage change in normalized virus concentration in the prior 15 days (hereafter referred to as *wastewater percentage change*), and percentage of wastewater samples with detectable virus in the prior 15 days. Our analysis focused on the first 2 NWSS-produced measures.

Because the wastewater percentile metric compares current with historical peak viral concentrations and because we aimed to compare data across sites, we restricted our analysis to sewersheds with available data in January 2022 (eFigure 1 in Supplement 1). Counties had to have submitted at least 1 week of data in January 2022 and have data available for more than 50% of the subsequent weeks to be further included in the analysis (eFigure 2 in Supplement 1). Implementing these criteria minimized fluctuation in percentile metric by date of assessment (eFigure 3 in Supplement 1 demonstrates similar percentile metrics for an example county for assessments in August vs October 2022).

We also restricted data to samples obtained from a treatment plant itself, rather than from pretreatment plant wastewater. In counties with more than 1 sewershed reporting to NWSS, we aggregated data from each sewershed to county level by creating a weighted average using each sewershed’s population served. Thus, the sewershed serving the largest population contributed the largest weight to the averaged county estimate.

#### Case and Hospitalization Rates

In the primary analysis, we used 2 CDC community level indicators as our dependent variables: reported new COVID-19 cases per 100 000 and new inpatient admissions per 100 000.^14^ We used publicly available time series data on aggregated counts of COVID-19 cases from state and local health departments, and hospital admissions from US Department of Health and Human Services Unified Hospital Data Surveillance System.^15,16^ Consistent with CDC reporting practices, we computed aggregate counts of COVID-19 cases and hospitalizations per 100 000 population from the past 7 days at the midpoint of each week. When comparing wastewater metrics to hospitalizations, we lagged new inpatient admissions by 2 weeks. We defined high COVID-19 community level using CDC-defined thresholds: (1) reported case rate equal to or greater than 200 new COVID-19 cases per 100 000 population, and (2) reported hospitalization rate equal to or greater than 10 new inpatient admissions per 100 000 population.

### Statistical Analysis

We grouped data by calendar quarters (January to March, April to June, and July to September). We obtained county population data from the 2021 US Census.^17^ To visually evaluate the association of our 2 wastewater metrics with clinical case metrics, we graphed these for 2022 for the most populous county from each US Census region. We also graphed the absolute wastewater concentrations within the county to visualize its association with the county-level wastewater metrics.

We then computed the sensitivity, specificity, and area under the receiver operating characteristic (AUC) by time period of wastewater metrics in identifying CDC thresholds for high COVID-19 case and hospitalization rates. We treated the wastewater metrics as the test of interest and the thresholds of cases and hospitalizations as comparative indicators of high COVID-19 community level. To determine whether combining both wastewater metrics was associated with high COVID-19 community levels, we used logistic regression accounting for wastewater percentile, percentage change, and the interaction of the 2 variables. Because our goal was to evaluate potential thresholds for high infection prevalence, we compared model predictive ability across probability cutoffs in which the sum of sensitivity and specificity was maximized (Youden index).^18^

In a sensitivity analysis, we evaluated wastewater percentile performance in small vs large counties; large counties are defined as population equal to or greater than 500 000 by US Census Bureau.^17^ We also conducted sensitivity analysis to test the association of current cases and hospitalization rates with rates of cases and hospitalizations lagged by 2 weeks, above CDC thresholds as our dependent variables.

Each county contributed data to the analysis for weeks during which wastewater data were available. Because not all counties consistently reported values, the number of counties included in the analysis varied per week. We assessed the association of the wastewater with clinical case metrics for each week stratified by calendar quarter. We computed bootstrapped 95% CIs for performance estimates using the bootstraps function. To account for within-county correlations in time series data, we performed resampling by county. Bootstrapped 95% CIs for figures were generated by calculating sensitivity at given specificity points using the ci.se function in R statistical software version 4.2.2 (R Project for Statistical Computing). We used R statistical packages epiR, rsample, and pROC to perform the analyses.

## Results

**Figure 1.**
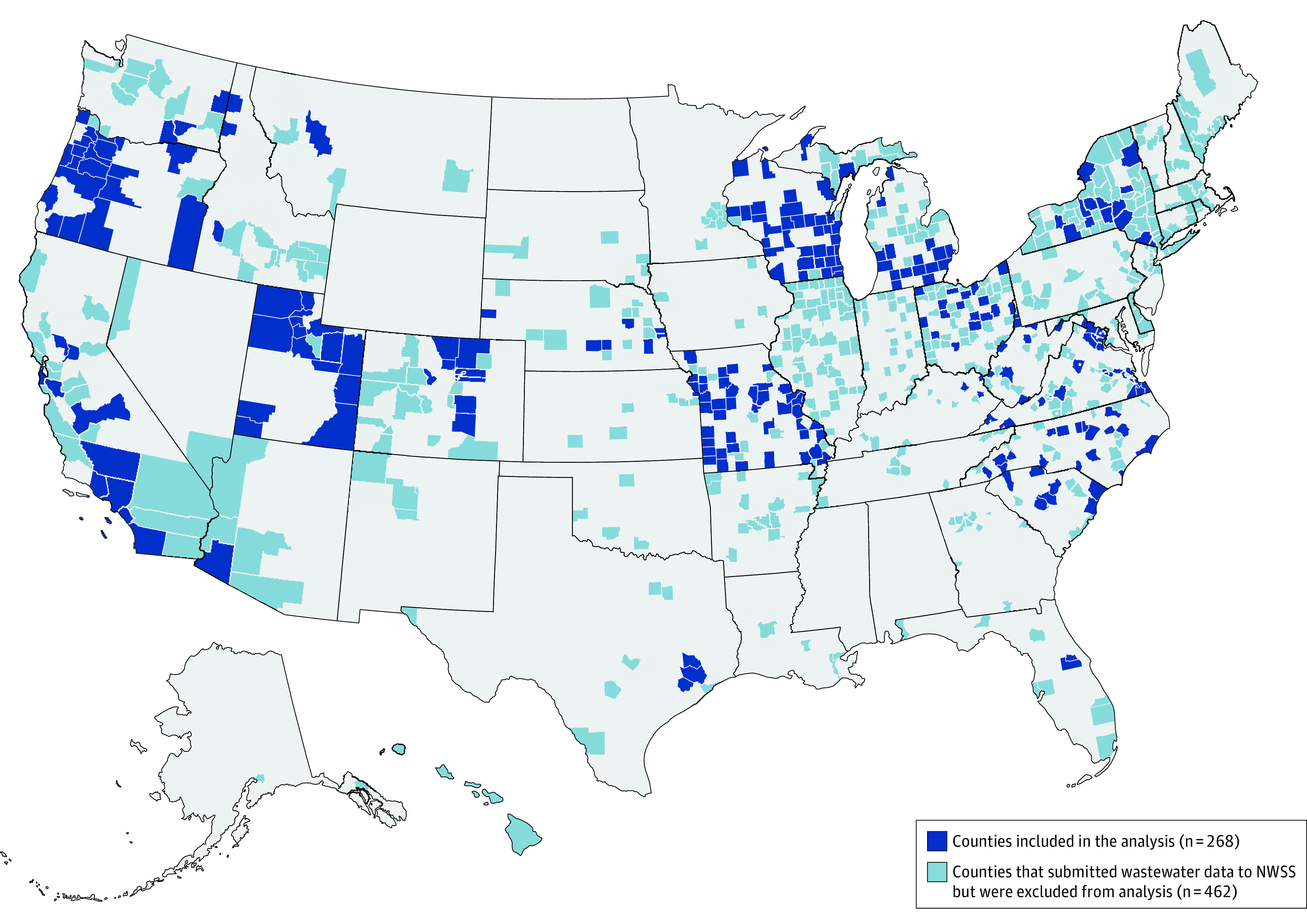
US Counties Submitting Wastewater Surveillance Data to the National Wastewater Surveillance System (NWSS) Between January 1, 2022, and September 30, 2022 Regions mapped in dark blue show counties included in the analysis (n = 268). Regions mapped in light blue show counties that submitted the wastewater data to NWSS, but were excluded from analysis (n = 462).

Among 730 wastewater treatment counties that submitted the analyzed metrics to NWSS during our study period, 268 counties across 22 states met our inclusion criteria (Figure 1 and eFigure 2 in Supplement 1). The median (IQR) population of counties included in the analysis was 95 938 (44 697-294 772) residents (Table). Comparatively, the overall US county population median (IQR) is 25 752 (10 818-67 899) residents. Consistent with national data, reported new case and hospitalization incidence rates were high in the first and third quarters, and lower in the second. Also consistent with national data, in our sampled counties, new case incidence was at its highest since the start of the pandemic in the first quarter of 2022 (eFigure 1 in Supplement 1).

**Table.** Sampled County Population and Sewershed Data, and Reported Case and Hospitalization Rates by Calendar Quarters of 2022

Variable	Counties (n = 268)^a^	Reported new cases, median (IQR), No./100 000 population	Reported new hospitalizations, median (IQR), No./100 000 population
Total county population, median (IQR), No. of residents	95 938 (44 697-294 772)	NA	NA
Population served by sampled sewersheds, median (IQR), No. of residents	49 831 (14 292-221 250)	NA	NA
Sampled sewersheds per county, median (IQR), No.	1 (1-2)	NA	NA
Weeks with available data, median (IQR)^b^	39 (38-39)	NA	NA
Calendar quarters of 2022			
January to March	NA	246 (73-928)	12 (4-28)
April to June	NA	132 (60-211)	5 (2-9)
July to September	NA	180 (129-238)	10 (5-16)

Abbreviation: NA, not applicable.

^a^ The number of counties for quarter 3 (July-September) was 263.

^b^ The total study period from January 1, 2022, through September 30, 2022, spanned 39 calendar weeks.

**Figure 2.**
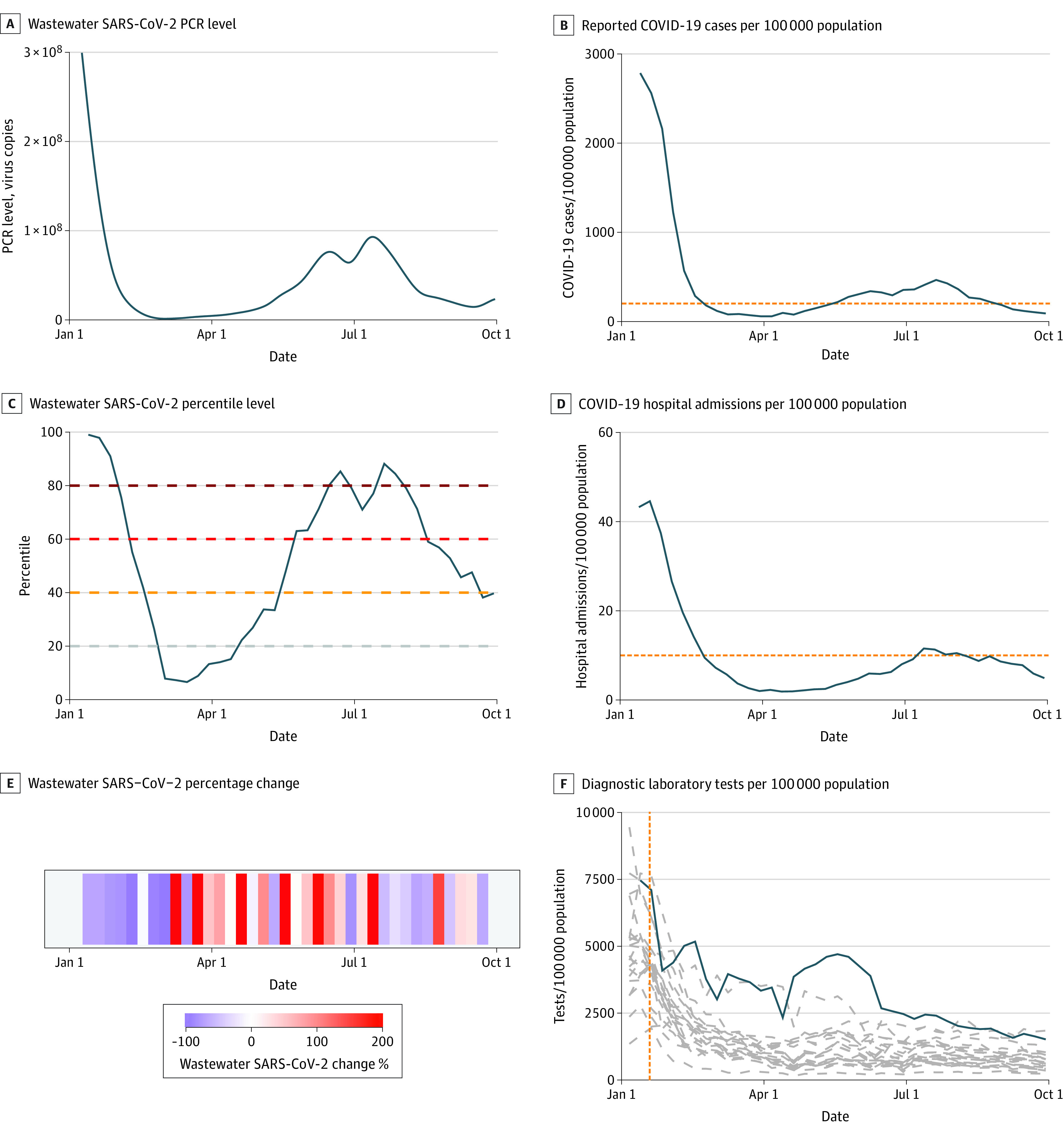
Time History of Wastewater Surveillance Data and Clinical Case Metrics From Los Angeles County, California, January 2022 and September 2022 A, Graph shows smoothed spline-fit polymerase chain reaction (PCR) concentrations of SARS-CoV-2 for each sampling location as reported by the Centers for Disease Control and Prevention National Wastewater Surveillance System. B, Graph shows reported COVID-19 cases per 100 000 population. C, Graph shows wastewater SARS-CoV-2 percentile level. D, Graph shows COVID-19 hospital admissions per 100 000 population. Horizontal dashed lines in B and D show thresholds for high COVID-19 community level (reported COVID-19 case rate ≥200 per 100 000 population and reported hospitalization rate ≥10 new inpatient admissions per 100 000 population, respectively). E, Graph shows wastewater SARS-CoV-2 15-day percentage change. F, Graph shows state-level data for diagnostic laboratory tests per 100 000 population (solid black line shows reported tests from the state of California; dashed gray blue show estimates for all other US states; dashed vertical orange line represents the date when distribution of rapid home tests was announced by the Biden administration, January 19, 2022). The solid blue lines in panels A, B, C, D, and F show weighted mean values using each sewershed’s population served. Data for the most populous counties in US Census regions Midwest and Northeast are shown in eFigure 4 in Supplement 1.

**Figure 3.**
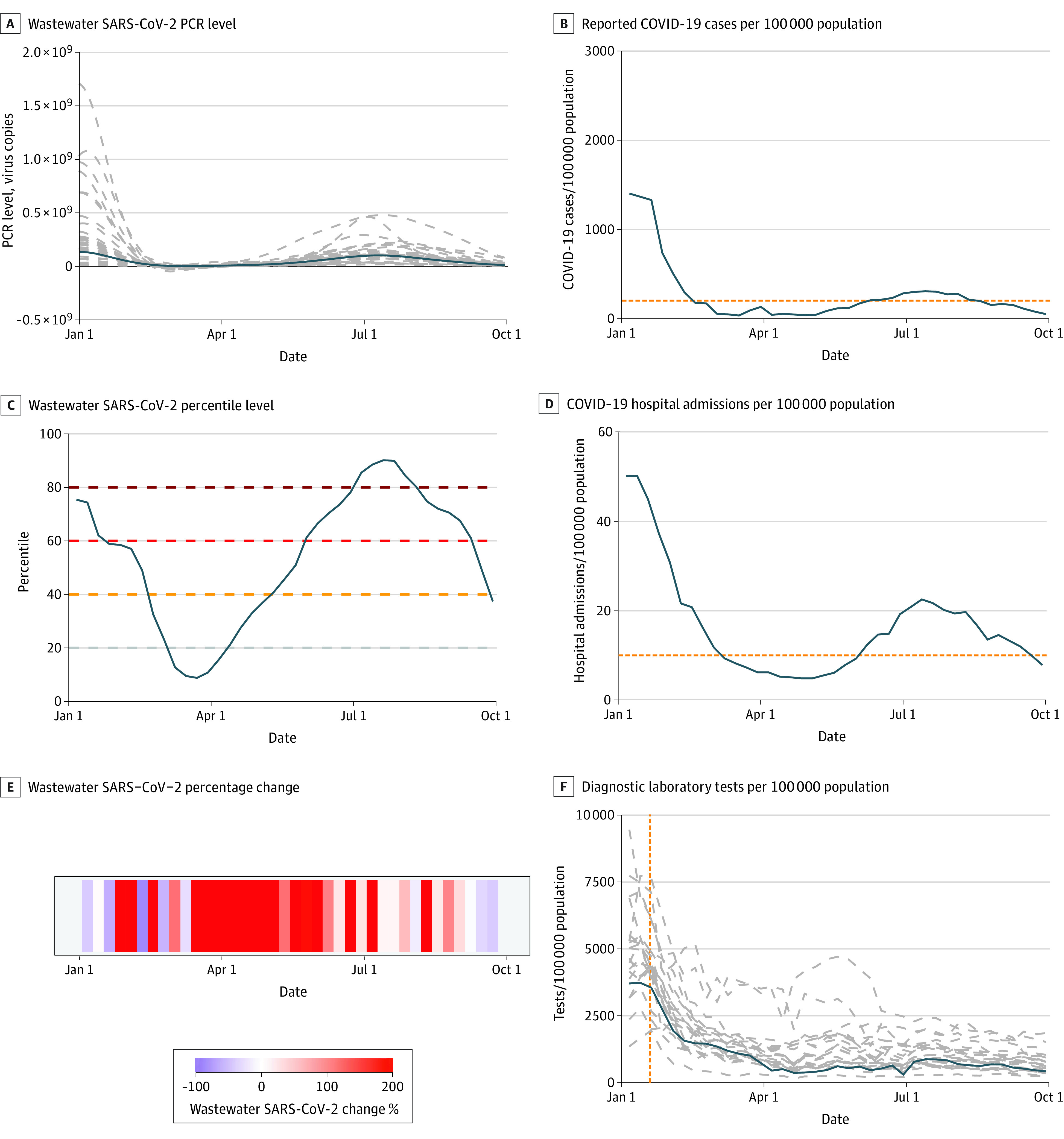
Time History of Wastewater Surveillance Data and Clinical Case Metrics From Harris County, Texas, January 2022 and September 2022 A, Graph shows smoothed spline-fit polymerase chain reaction (PCR) concentrations of SARS-CoV-2 for each sampling location as reported by the Centers for Disease Control and Prevention National Wastewater Surveillance System. When multiple sewersheds were sampled within a county, dashed gray lines in panel A represent individual sewersheds. B, Graph shows reported COVID-19 cases per 100 000 population. C, Graph shows wastewater SARS-CoV-2 percentile level. D, Graph shows COVID-19 hospital admissions per 100 000 population. Horizontal dashed lines in B and D show thresholds for high COVID-19 community level (reported COVID-19 case rate ≥200 per 100 000 population and reported hospitalization rate ≥10 new inpatient admissions per 100 000 population, respectively). E, Graph shows wastewater SARS-CoV-2 15-day percentage change. F, Graph shows state-level data for diagnostic laboratory tests per 100 000 population (solid blue lines show reported tests from the state of Texas; dashed gray lines show estimates for all other US states; dashed vertical orange line represents the date when distribution of rapid home tests was announced by the Biden administration, January 19, 2022). The solid blue lines in panels A, B, C, D, and F show weighted mean values using each sewershed’s population served. Data for the most populous counties in US Census regions Midwest and Northeast are shown in eFigure 4 in Supplement 1.

**Figure 4.**
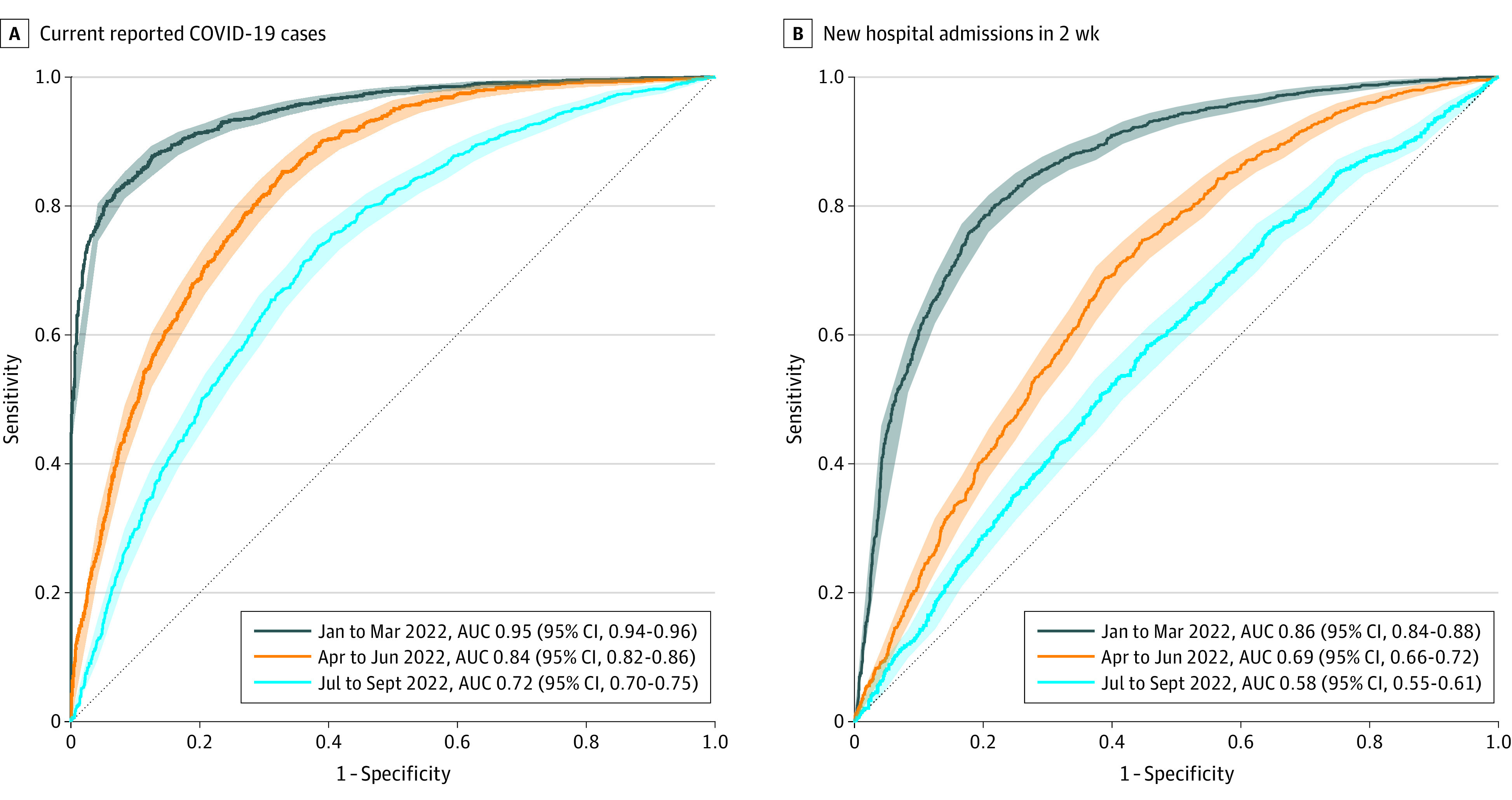
Performance of Wastewater Percentile in Reference to Clinical Case Metrics Stratified by Calendar Quartile of 2022 Graphs show areas under the curve (AUCs) of wastewater percentile in reference to current reported COVID-19 cases (≥200 per 100 000 population) (A) and new hospital admissions in 2 weeks (≥10 per 100 000 population) (B). Shaded ribbons show bootstrapped 95% CIs for sensitivity at given specificity.

Plots of the available data for 2022 from the most populous counties in each US Census region demonstrate a direct association between wastewater percentile and absolute SARS-CoV-2 concentrations (Figure 2 and Figure 3; eFigure 4 in Supplement 1). The 15-day percentage change variable fluctuated widely (eTable in Supplement 1). In the first quarter of 2022, facility wastewater percentile was closely associated with cases and hospitalizations. In contrast, the association was less evident in the third quarter when reported case and hospitalization rates were low, despite high levels of SARS-CoV2 in wastewater as indicated by facility percentile. In AUC analyses incorporating data from all counties, wastewater percentile was closely associated with high reported case (>200 per 100 000; AUC, 0.95; 95% CI, 0.94-0.96) and hospitalization rates (>10 per 100 000 lagged by 2 weeks; AUC, 0.86; 95% CI, 0.84-0.88) in the first quarter of 2022 (Figure 4).

Among counties sharing wastewater data to NWSS since January 2021, wastewater percentile of 51% was the Youden index threshold for detecting cases exceeding 200 per 100 000 population (sensitivity, 0.82; 95% CI, 0.80-0.84; specificity, 0.93; 95% CI, 0.92-0.95) in the first quarter of 2022. Similarly, wastewater percentile of 54% was the Youden index threshold hospitalizations exceeding 10 per 100 000 population (sensitivity, 0.80; 95% CI, 0.77-0.83; specificity, 0.78; 95% CI, 0.76-0.81). In the overall analysis, AUC declined over the next 2 quarters (second quarter AUC, 0.84; 95% CI, 0.82-0.86; third quarter AUC, 0.72; 95% CI, 0.70-0.75) for the association of wastewater percentile with cases and hospitalizations (Figure 4). Performance was similar in small and large counties: AUCs for the first quarter were 0.95 (95% CI, 0.94-0.96) for cases in small counties, 0.95 (95% CI, 0.93-0.98) for cases in large counties, 0.85 (95% CI, 0.82-0.87) for hospitalizations in small counties, and 0.94 (95% CI, 0.91-0.97) for hospitalizations in small large counties (eFigures 5 and 6 in Supplement 1).

The percentage change metric performed poorly, with AUCs ranging from 0.51 (95% CI, 0.50-0.53) to 0.57 (95% CI, 0.55-0.59) for reported new cases, and from 0.50 (95% CI, 0.48-0.52) to 0.55 (95% CI, 0.53-0.57) for hospitalizations across the 3 quarters (eFigure 7 in Supplement 1). Combining wastewater facility percentile, percentage change, and the interaction of the 2 variables in a logistic regression analysis yielded estimates of predictive performance similar to those found using the percentile metric alone (eFigure 8 in Supplement 1). AUC analyses examining the performance of clinical metrics as predictors of future clinical outcomes (ie, current case and hospitalization rates’ correlation with case and hospital admission rates lagged by 2 weeks) indicated an association (eFigures 9 and 10 in Supplement 1) for both clinical metrics in the first quarter of 2022. As with wastewater percentile, however, performance declined over the next 2 quarters.

## Discussion

To our knowledge, this cohort study with a time series analysis is the first to examine CDC-generated wastewater metrics from sewersheds located throughout the nation. We observed a direct association of a county’s SARS-CoV-2 wastewater concentration, relative to its maximal observed, with COVID-19 cases and hospitalizations for US counties during the first quarter of the 2022. When little home testing was being conducted, wastewater percentiles in all counties tracked quite closely with new cases per 100 000 population. However, the association of the wastewater percentile with COVID-19 reported cases decreased over the next 2 quarters, indicating an increasing dissociation between community viral prevalence and reports of infection to health departments. There was also increasing dissociation between wastewater and new hospitalization rates, perhaps indicative of lower rates of COVID-19–related hospitalization with heightened population immunity due to prior infection and vaccination, potentially lower virulence of evolving strains, and/or reduction in routine admissions testing in hospitals.

In the first quarter of 2022, we evaluated wastewater metrics against case metrics when many wastewater facilities were contributing data nationwide and when at-home testing, while increasing, was still not ubiquitous.^19^ No nationally representative data exist on the relative use of home tests vs laboratory or point-of-care tests in the US, but from a large internet survey of more than 450 000 US adults, among persons with symptoms, there was a 4-fold increase in report of home COVID-19 tests, from 5% in the Delta-dominant period in fall 2021 to 20% in the Omicron-dominant period in winter 2022.^19^ Results of home testing are rarely reported. In a recent analysis of self-testing data from October 2021 to May 2022, Ritchey et al^20^ reported that results from only 3% of the nearly 400 million at-home tests produced by 4 US manufacturers were voluntarily reported to health authorities. Because we tracked the same facilities in the same counties over the subsequent 2 quarters, we postulate that it is the increasing use of at-home testing, rather than any changes in the association of SARS-CoV-2 incidence with shedding into the wastewater system, that led to our observed decline in the performance of wastewater percentile in detecting new cases.

In our analysis, the benchmark for evaluating data from facilities sharing data for at least 6 months is critical for interpretation, because the wastewater percentile metric places all newer data relative to the highest community prevalence of COVID-19 seen in the county. As more wastewater facilities come online, benchmarking remains a critical unresolved question. There are several ways to address lack of historical data, including imputation models and adopting references relative to data from neighboring established facilities. Furthermore, should there a surge in absolute viral concentration far exceeding that observed during Omicron variant circulation in 2022, the percentile metric may require recalibration or reassessment for thresholds correlating with high infection prevalence. Other potential improvements, such as selecting sewersheds that are better representative of counties sampled, could increase the yield of a sentinel surveillance system.

Since fall 2020, the CDC has invested over $100 million to support wastewater surveillance infrastructure in the US, with the largest share of the investment occurring in August 2022.^21^ Although the utility of wastewater surveillance extends beyond COVID-19, national level data are collated and reported only for COVID-19. The NWSS makes substantial efforts to convert absolute viral concentration data into comparable measures across and within sites. As of September 2022, 1213 facilities representing 741 counties and 50 states were submitting data, which were updated weekly.^13^ In our analysis, more than 99% of analyzed facilities had more than half of weeks covered from the past 3 quarters. Thus, timely nationwide data are available for an increasing number of US residents. Although a few counties publish and publicize their results^22^ to raise public awareness, inform mask wearing, and promote social distancing, we lack a national strategy for the use of wastewater surveillance. If we presume the first quarter association we observed between wastewater percentile and new cases likely holds steady, then a wastewater percentile of 51% of maximum as generated by the NWSS can reflect high infection prevalence, regardless of reported case counts.

As a counterpoint, as COVID-19 infection evolves clinically for the largest share of the population, for whom there is a potentially lower risk for hospitalization and death with vaccination and the Omicron subvariants, some could argue that investments in capturing true prevalence of circulating disease are unnecessary. This is a reasonable trade-off to consider, but it needs to be contextualized with 2 important points. First, medically vulnerable populations, such organ transplant recipients,^23^ persons receiving chemotherapy,^24^ and persons receiving dialysis,^25^ are suboptimally protected by vaccinations and remain at high risk for adverse health outcomes from COVID-19 infection. Thus, awareness of true disease prevalence could promote additional protective measures tailored to these populations and enable earlier treatment. For example, during periods of high disease circulation, universal asymptomatic testing could be offered in long-term care facilities, with nirmatrelvir-ritonavir treatment provided early to patients testing positive. Second, even among the general population, COVID-19 infection or reinfection has been shown to be associated with adverse health events, including symptoms of post–COVID-19 condition and hospitalizations. Moreover, there remain risks of waning immunity and worsening variants. In scenarios where home-based testing and underreporting of cases are common, wastewater surveillance may also enable better measurement of virulence by quantifying the denominator for the numerator of case counts.

Our research on wastewater was done during a period of marked flux in diagnostics, vaccination, and disease acuity. We postulate that wastewater surveillance is the most consistent measure of infection prevalence during this unstable time, especially given its sensitivity even in low-prevalence settings.^26^ However, infection prevalence does not reflect disease acuity, and this may, in part, explain the smaller association of both wastewater percentile and case rates with high hospitalization rates (lagged by 2 weeks) in the second and third quarter of 2022. As vaccination, antiviral treatment, increasing population immunity, and changes in variants made infection less severe, fewer patients accessed diagnostic testing and were hospitalized despite continued shedding into the sewersheds. It is our expectation that, as COVID-19 settles into endemicity, decisions to test and report will reach a steady state (ie, that a stable proportion of cases will be tested and reported). When this happens, public health officials will be able to estimate total cases from reported cases. Notably, however, as of May 2023, the CDC has discontinued collated public sharing of COVID-19 cases by county but continues to update NWSS data. Furthermore, in a future pandemic, until stable testing rates are achieved and sufficient data on infection virulence are gathered, wastewater surveillance may be the best early signal of a local outbreak and as a method to monitor circulating variants.^27,28,29^

We evaluated wastewater metrics against 2 commonly used outcomes of case and hospitalization rates and conclude that wastewater metrics likely provide the better estimate of infection prevalence as formal case testing declines. In the future, complementary measures, such as school attendance^30^ or emergency department visits for influenza-like illness, could be integrated with wastewater metrics to better understand the clinical and public health impact of wastewater metrics.

### Limitations

Our analysis is limited by the need to rely on a subset of facilities with sufficient data to not only track back to a true community peak, but also to allow a relatively stable percentile value assigned to an absolute viral concentration over time. Newer facilities may experience substantial fluctuations in the association of absolute viral concentration with assigned percentile, unless they benchmark to a neighboring county reference and/or use imputed historical data. To facilitate potential public health adoption, we also only evaluated metrics available within NWSS, rather than generating de novo metrics using raw or normalized wastewater data. The counties in our wastewater cohort are larger than the average US county. Two factors may explain this. First, smaller counties may have fewer personnel or resources to devote to wastewater surveillance. Second, smaller counties are more likely rural, and households in rural counties are more likely to rely on individual septic systems rather than publicly owned treatment works. Although rural counties make up 97% of the landmass of the US and 63% of US counties, only 14% of the US population lives in these areas.^31^ This important subset, however, is missed by wastewater surveillance that relies on public wastewater treatment works.

## Conclusions

In summary, in this first analysis of wastewater metrics for SARS-CoV-2 incorporating data from the breadth of public health–monitored sewersheds in the US, found find that counties conducting wastewater surveillance and reporting data to the CDC NWSS in the US could use an aggregated measure of the percentage of maximum wastewater SARS-CoV-2 concentration to estimate county-level prevalence of COVID-19. Counties with a longer historical data record, tracking back to at least January 2022, will generally provide the most reliable estimates. We demonstrated that wastewater surveillance can be operationalized to fulfill the relevant public policy goals of public awareness of true SARS-CoV-2 incidence and implementation of additional actions specifically designed to protect medically vulnerable populations.

## Notes

Supplement 1. **eTable.** Sampled County Population and Sewershed Data, and Case and Hospitalization Rates by Quarters of 2022 **eFigure 1. **Diagnostic Testing and Reported New COVID-19 Cases in the US Between March 1, 2020, and September 30, 2022 **eFigure 2.** Selection of Counties Included in Analysis **eFigure 3.** Changes in Wastewater Percentile Value as New Data Becomes Available, Wayne County, Michigan, as an Illustrative Example **eFigure 4.** Time History of Wastewater Surveillance Data and Clinical Case Metrics From the Most Populous Counties in US Census Regions Midwest and Northeast Between January 2022 and September 2022 **eFigure 5.** Performance of Wastewater Percentile in Reference to Clinical Case Metrics in Small US Counties (n=230) Stratified by Calendar Quartile of 2022 **eFigure 6.** Performance of Wastewater Percentile in Reference to Clinical Case Metrics in Large US Counties (n=38) Stratified by Calendar Quartile of 2022 **eFigure 7.** Performance of 15-Day Wastewater Percent Change in Reference to Clinical Case Metrics Stratified by Calendar Quartile of 2022 **eFigure 8.** Performance of Combined Wastewater Metrics in Reference to Clinical Case Metrics Stratified by Calendar Quartile of 2022 **eFigure 9.** Performance of Current Reported COVID-19 Case Rates in Reference to Clinical Case Metrics Stratified by Calendar Quartile of 2022 **eFigure 10.** Performance of Current COVID-19 Hospital Admission Rate in Reference to Clinical Case Metrics Stratified by Calendar Quartile of 2022

Supplement 2.
Data Sharing Statement
