# Supplementary material for: Use of Wastewater Metrics to Track COVID-19 in the US
Source: JAMA Netw Open. 2023 Jul 26;6(7):e2325591. doi: 10.1001/jamanetworkopen.2023.25591 (PMC10372707; doi:10.1001/jamanetworkopen.2023.25591)
Supplement: Supplement 1. — eTable. Sampled County Population and Sewershed Data, and Case and Hospitalization Rates by Quarters of 2022 eFigure 1. Diagnostic Testing and Reported New COVID-19 Cases in the US Between March 1, 2020, and September 30, 2022 eFigure 2. Selection of Counties Included in Analysis eFigure 3. Changes in Wastewater Percentile Value as New Data Becomes Available, Wayne County, Michigan, as an Illustrative Example eFigure 4. Time History of Wastewater Surveillance Data and Clinical Case Metrics From the Most Populous Counties in US Census Regions Midwest and Northeast Between January 2022 and September 2022 eFigure 5. Performance of Wastewater Percentile in Reference to Clinical Case Metrics in Small US Counties (n=230) Stratified by Calendar Quartile of 2022 eFigure 6. Performance of Wastewater Percentile in Reference to Clinical Case Metrics in Large US Counties (n=38) Stratified by Calendar Quartile of 2022 eFigure 7. Performance of 15-Day Wastewater Percent Change in Reference to Clinical Case Metrics Stratified by Calendar Quartile of 2022 eFigure 8. Performance of Combined Wastewater Metrics in Reference to Clinical Case Metrics Stratified by Calendar Quartile of 2022 eFigure 9. Performance of Current Reported COVID-19 Case Rates in Reference to Clinical Case Metrics Stratified by Calendar Quartile of 2022 eFigure 10. Performance of Current COVID-19 Hospital Admission Rate in Reference to Clinical Case Metrics Stratified by Calendar Quartile of 2022 [file jamanetwopen-e2325591-s001.pdf]

Supplemental Online Content

Varkila MRJ, Montez-Rath ME, Salomon JA, et al. Use of wastewater metrics to track COVID-19 in the US. *JAMA Netw Open*. 2023;6(7):e2325591. doi:10.1001/jamanetworkopen.2023.25591

eTable. Sampled County Population and Sewershed Data, and Case and Hospitalization Rates by Quarters of 2022
eFigure 1. Diagnostic Testing and Reported New COVID-19 Cases in the US Between March 1, 2020, and September 30, 2022
eFigure 2. Selection of Counties Included in Analysis
eFigure 3. Changes in Wastewater Percentile Value as New Data Becomes Available, Wayne County, Michigan, as an Illustrative Example
eFigure 4. Time History of Wastewater Surveillance Data and Clinical Case Metrics From the Most Populous Counties in US Census Regions Midwest and Northeast Between January 2022 and September 2022
eFigure 5. Performance of Wastewater Percentile in Reference to Clinical Case Metrics in Small US Counties (n=230) Stratified by Calendar Quartile of 2022
eFigure 6. Performance of Wastewater Percentile in Reference to Clinical Case Metrics in Large US Counties (n=38) Stratified by Calendar Quartile of 2022
eFigure 7. Performance of 15-Day Wastewater Percent Change in Reference to Clinical Case Metrics Stratified by Calendar Quartile of 2022
eFigure 8. Performance of Combined Wastewater Metrics in Reference to Clinical Case Metrics Stratified by Calendar Quartile of 2022
eFigure 9. Performance of Current Reported COVID-19 Case Rates in Reference to Clinical Case Metrics Stratified by Calendar Quartile of 2022
eFigure 10. Performance of Current COVID-19 Hospital Admission Rate in Reference to Clinical Case Metrics Stratified by Calendar Quartile of 2022

This supplemental material has been provided by the authors to give readers additional information about their work.

© 2023 Varkila MRJ et al. *JAMA Network Open.*

eTable. Sampled County Population and Sewershed Data, and Case and Hospitalization Rates by Quarters of 2022

	Jan-Mar 2022 (n=268)					Apr-Jun 2022 (n=268)					Jul-Sep 2022 (n=263)				
	Median	Min	Max	25th	75th	Median	Min	Max	25th	75th	Median	Min	Max	25th	75th
Weeks with available data, number	13	1	13	12	13	13	4	13	13	13	13	8	13	13	13
Wastewater sites per county, number	1	1	33	1	2	1	1	33	1	2	1	1	33	1	2
Total county population,	95938	8376	9829544	44697	294772	95938	8376	9829544	44697	294772	96017	8376	9829544	45242	299647
County population served by sampled sewersheds	49831	3076	3500000	14292	221250	49831	3076	3500000	14292	221250	49662	3076	3500000	14410	198804
Wastewater percentile level, %	46.6	0	100	17.0	83.5	44.4	0	95.8	25.0	62.2	60.5	1.50	100.0	47.7	72.2
15-day percent change variable, %	-42	-100	2147483647	-80.0	51	30	-100	2147483647	-39	226	-8	-100	2147483647	-60	91
New cases per 100,000 population	245.8	3.0	4728.6	73.3	928.1	132.1	0	826.3	59.9	210.7	180.0	24.4	994.5	128.5	238.1
New hospitalizations per 100,000 population	11.5	0	291.7	4.2	27.7	4.8	0	70.9	2.0	9.3	9.6	0	124.2	5.4	15.5

© 2023 Varkila MRJ et al. *JAMA Network Open.*

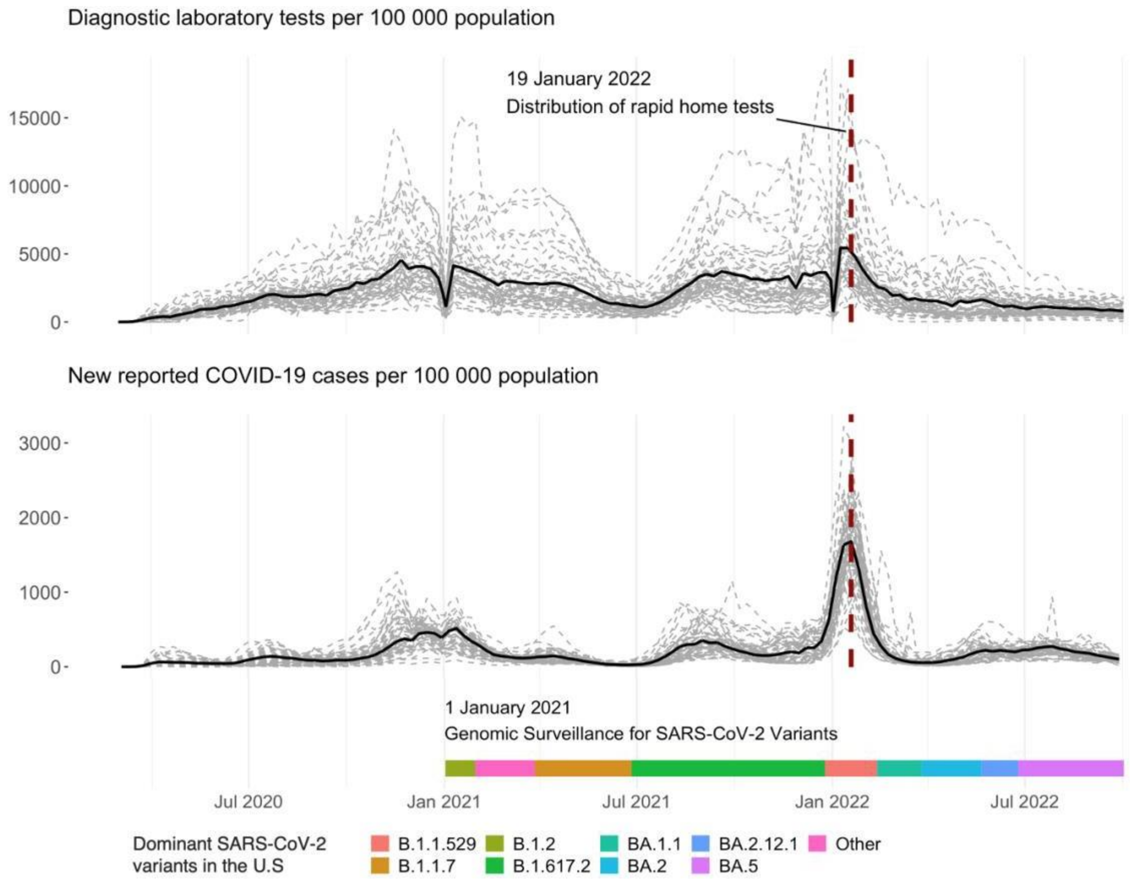

eFigure 1. Diagnostic Testing and Reported New COVID-19 Cases in the US Between March 1, 2020, and September 30. 2022

The dashed gray lines represent weekly totals of diagnostic laboratory tests (top) and new reported COVID-19 cases (bottom) per 100,000 population by state. The solid black line shows total numbers for the United States. The colored horizontal bars represent time periods during which specific SARS-CoV-2 variants were dominant in the United States reported by the CDC as of January 1 2021. The dashed vertical line shows the date when distribution of rapid home tests was announced by the Biden administration.

© 2023 Varkila MRJ et al. *JAMA Network Open.*

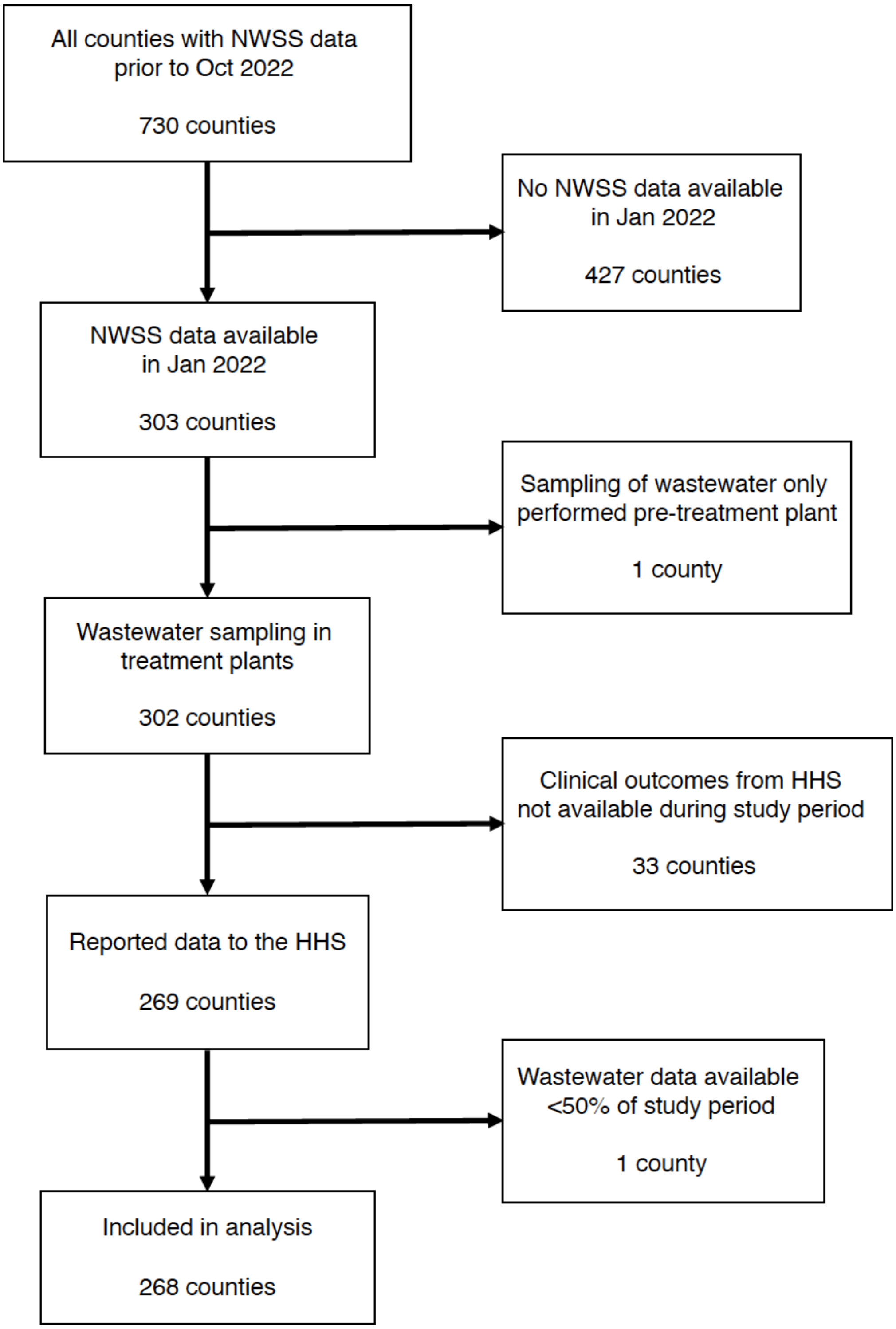

eFigure 2. Selection of Counties Included in Analysis

© 2023 Varkila MRJ et al. *JAMA Network Open.*

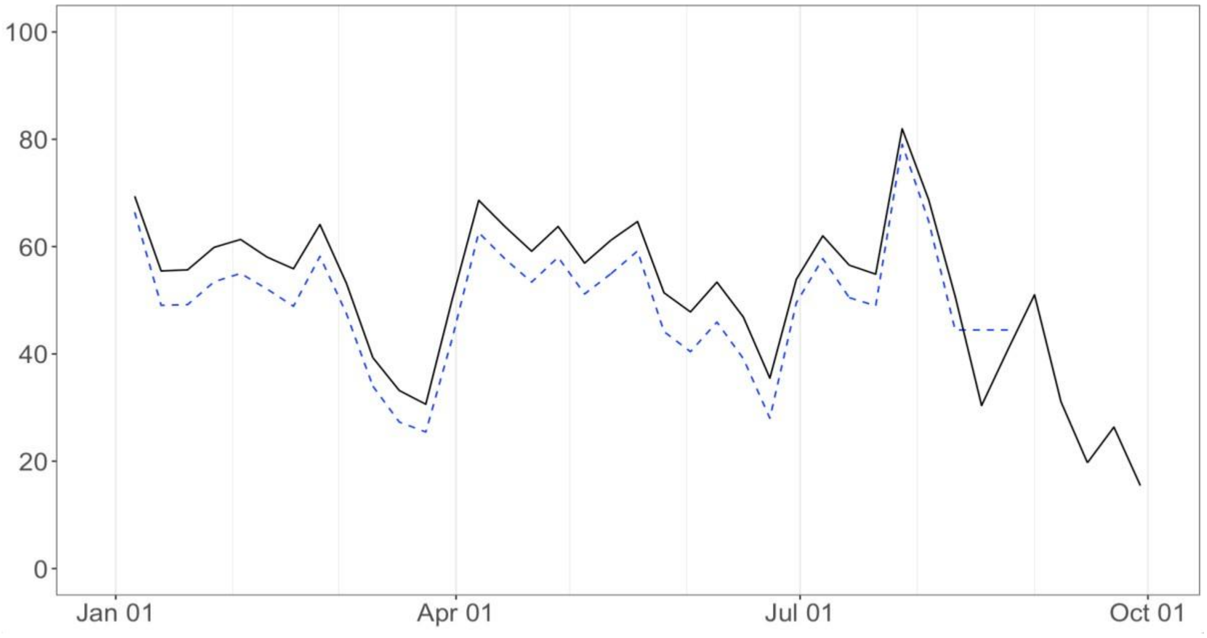

eFigure 3. Changes in Wastewater Percentile Value as New Data Becomes Available, Wayne County, Michigan, as an Illustrative Example

The blue dashed line represents wastewater percentile values from data downloaded in August 2022 and the black solid line represents values from data downloaded in October 2022.

© 2023 Varkila MRJ et al. *JAMA Network Open.*

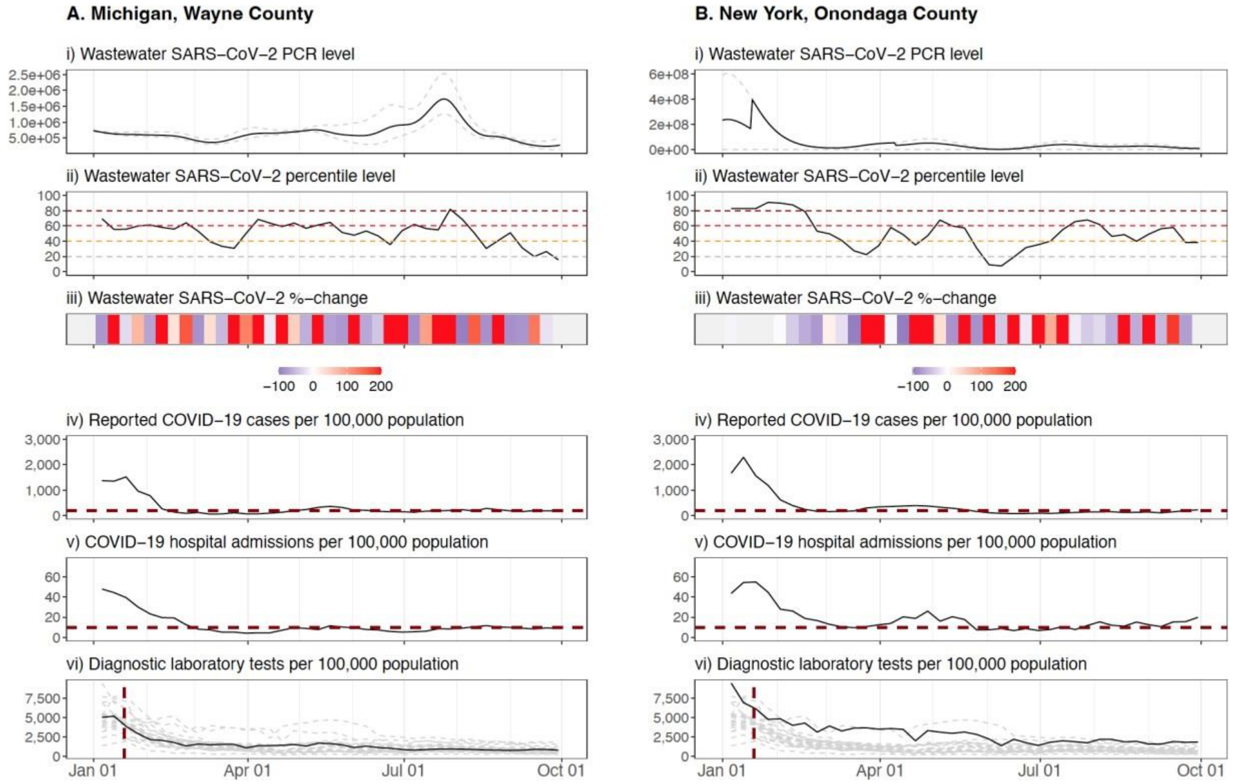

eFigure 4. Time History of Wastewater Surveillance Data and Clinical Case Metrics From the Most Populous Counties in US Census Regions Midwest and Northeast Between January 2022 and September 2022

Data are shown for the most populous counties in U.S. Census regions (A) Midwest: Wayne County, Michigan, and (B) Northeast: Onondaga County, New York. Panel (i) shows smoothed spline-fit PCR concentrations of SARS-CoV-2 for each sampling location as reported by the CDC NWSS. When multiple sewersheds were sampled within a county, dashed grey lines in panel (i) represent individual sewersheds. The solid black lines in panels (i), (ii) wastewater SARS-CoV-2 percentile level, and (iii) wastewater SARS-CoV-2 15-day percent change show weighted mean values using each sewershed’s population served. Horizontal dashed lines in panels (iv) and (v) show thresholds for high COVID-19 community level (reported COVID-19 case rate equal to or greater than 200 per 100,000 population and reported hospitalization rate equal to or greater than 10 new inpatient admissions per 100,000 population, respectively). Panel (vi) shows state-level data (solid black lines show reported tests from the state of Michigan (A), and New York (B); dashed grey lines show estimates for all other U.S. states).

© 2023 Varkila MRJ et al. *JAMA Network Open.*

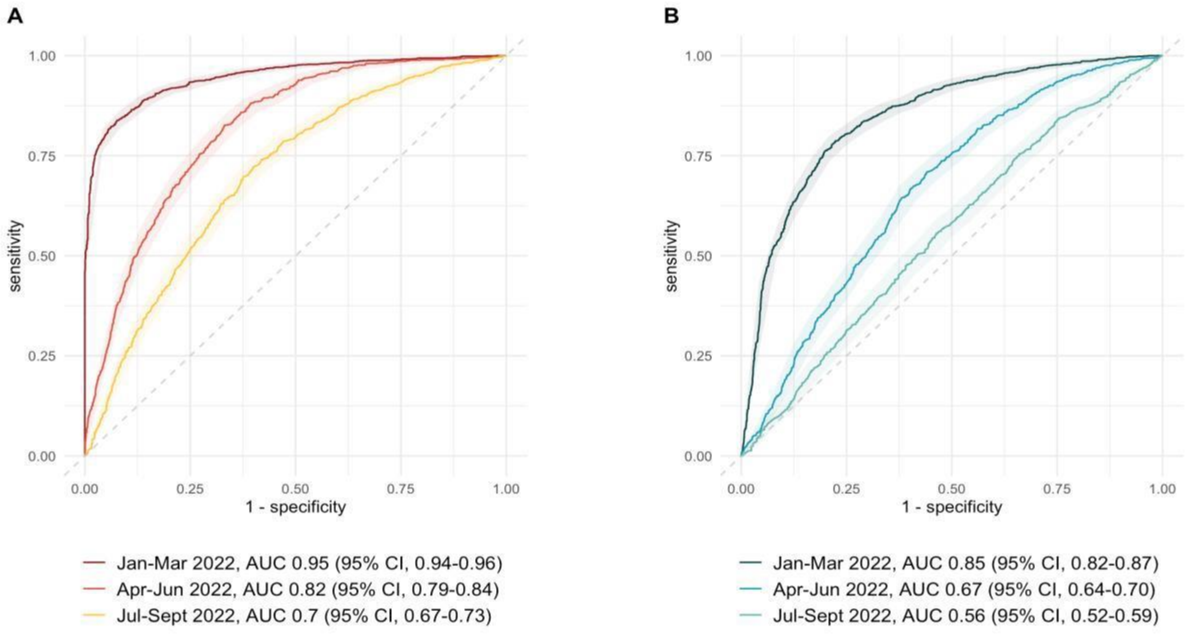

eFigure 5. Performance of Wastewater Percentile in Reference to Clinical Case Metrics in Small US Counties (n=230) Stratified by Calendar Quartile of 2022

AUC of wastewater percentile in reference to (A) Current reported COVID-19 cases (≥200 per 100,000 population), (B) New hospital admissions in two weeks (≥10 per 100,000 population). Small counties were defined as counties with a total population less than 500,000 according to the 2021 U.S. Census.

© 2023 Varkila MRJ et al. *JAMA Network Open.*

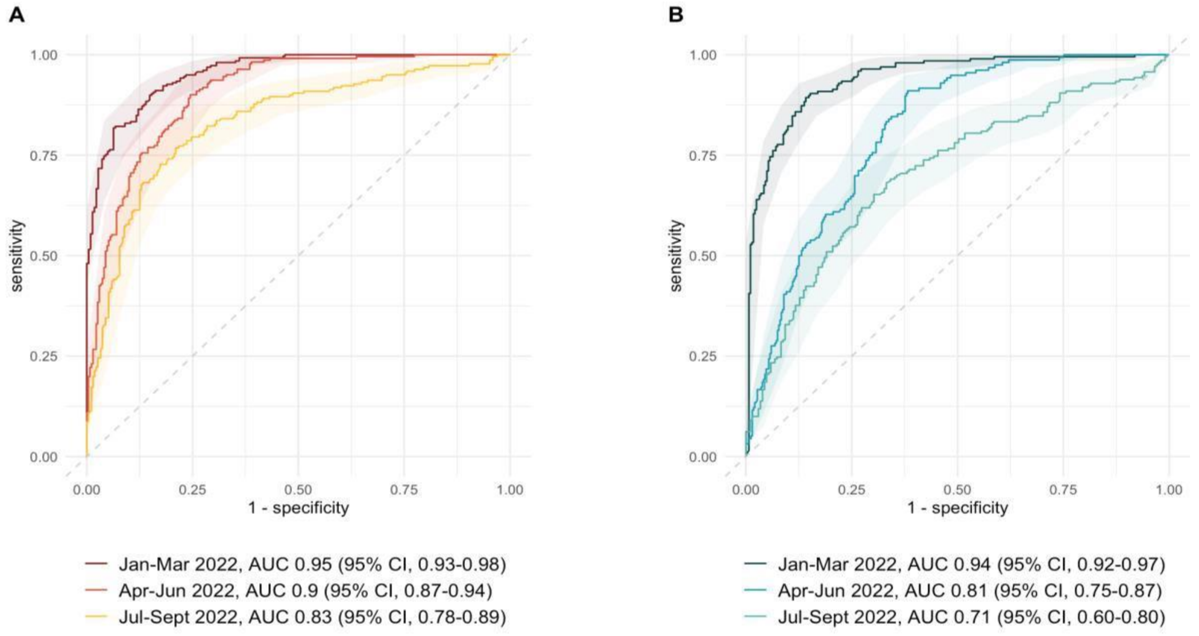

eFigure 6. Performance of Wastewater Percentile in Reference to Clinical Case Metrics in Large US Counties (n=38) Stratified by Calendar Quartile of 2022

AUC of wastewater percentile in reference to (A) Current reported COVID-19 cases (≥200 per 100,000 population), (B) New hospital admissions in two weeks (≥10 per 100,000 population). Large counties were defined as counties with a total population equal to or greater than 500,000 according to the 2021 U.S. Census.

© 2023 Varkila MRJ et al. *JAMA Network Open.*

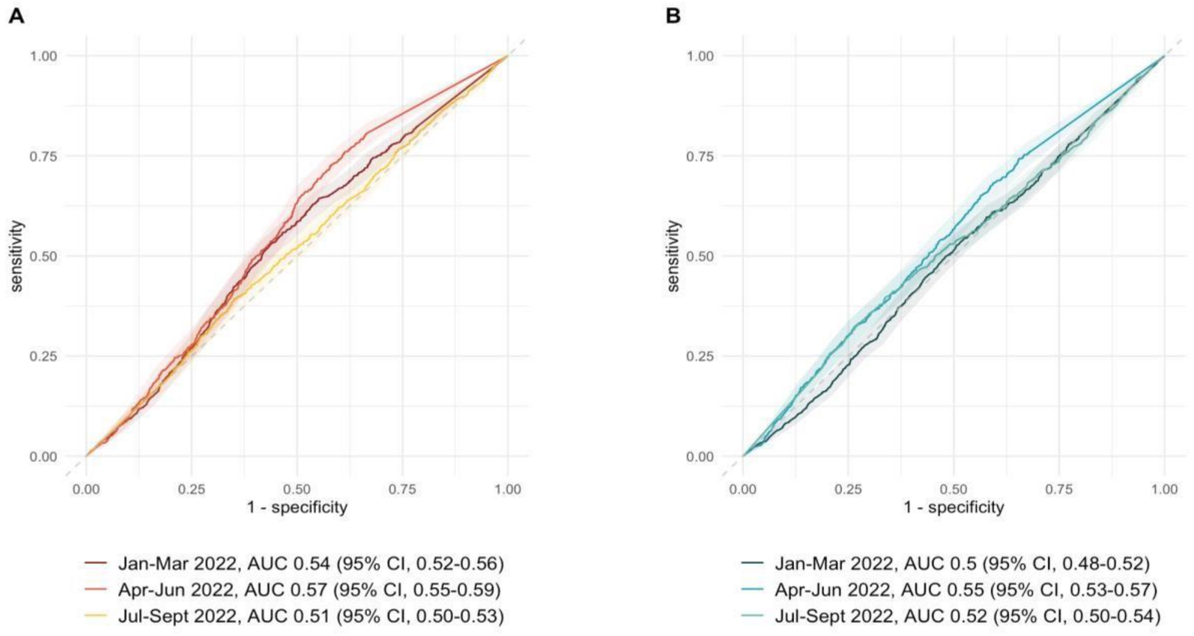

eFigure 7. Performance of 15-Day Wastewater Percent Change in Reference to Clinical Case Metrics Stratified by Calendar Quartile of 2022

AUC of 15-day wastewater percent change in reference to (A) Current reported COVID-19 cases (≥200 per 100,000 population), (B) New hospital admissions in two weeks (≥10 per 100,000 population). @

© 2023 Varkila MRJ et al. *JAMA Network Open.*

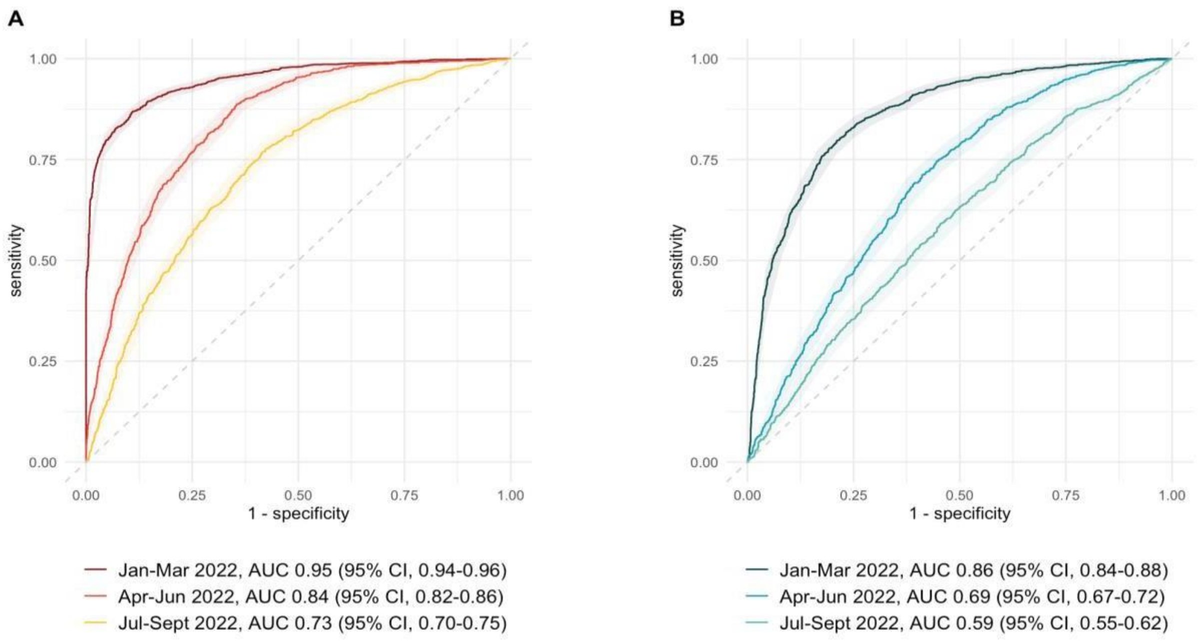

eFigure 8. Performance of Combined Wastewater Metrics in Reference to Clinical Case Metrics Stratified by Calendar Quartile of 2022

AUC of combined wastewater metrics in reference to (A) Current reported COVID-19 cases (≥200 per 100,000 population), (B) New hospital admissions in two weeks (≥10 per 100,000 population). To estimate combined effects of wastewater metrics we used logistic regression accounting for wastewater percentile, percent change, and the interaction of the two.

© 2023 Varkila MRJ et al. *JAMA Network Open.*

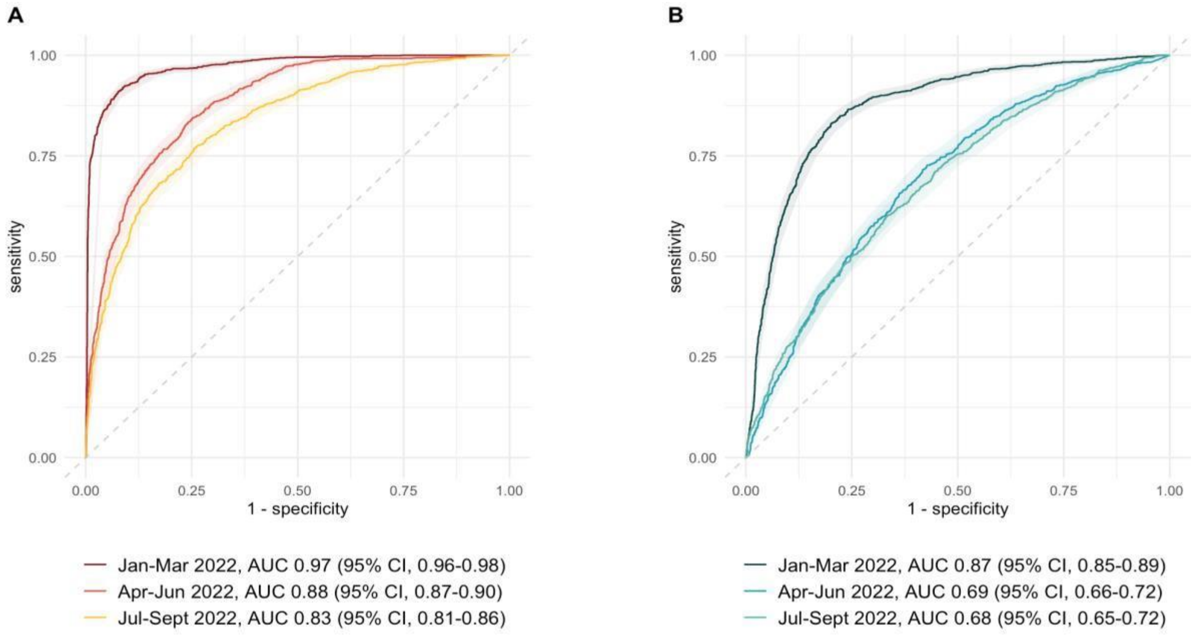

eFigure 9. Performance of Current Reported COVID-19 Case Rates in Reference to Clinical Case Metrics Stratified by Calendar Quartile of 2022

AUC of current reported case rates in reference to (A) reported COVID-19 cases in two weeks (≥200 per 100,000 population), (B) New hospital admissions in two weeks (≥10 per 100,000 population).

© 2023 Varkila MRJ et al. *JAMA Network Open.*

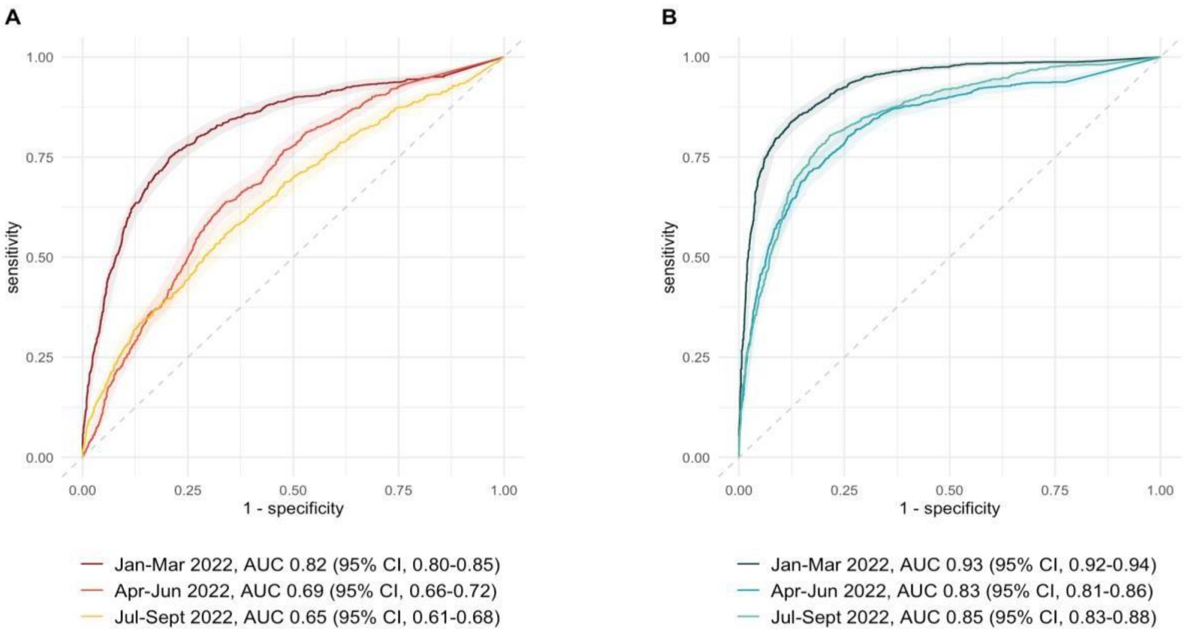

eFigure 10. Performance of Current COVID-19 Hospital Admission Rate in Reference to Clinical Case Metrics Stratified by Calendar Quartile of 2022

AUC of current hospitalization rates in reference to (A) reported COVID-19 cases in two weeks (≥200 per 100,000 population), (B) New hospital admissions in two weeks (≥10 per 100,000 population).

© 2023 Varkila MRJ et al. *JAMA Network Open.*
