# Supplementary material for: Use of Wastewater Metrics to Track COVID-19 in the US
Source: JAMA Netw Open. 2023 Jul 26;6(7):e2325591. doi: 10.1001/jamanetworkopen.2023.25591 (PMC10372707; doi:10.1001/jamanetworkopen.2023.25591)
Supplement: Supplement 2. — Data Sharing Statement [file jamanetwopen-e2325591-s002.pdf]

Data Sharing Statement

Varkila. Use of Wastewater Metrics to Track COVID-19 in the US. *JAMA Netw Open*.
Published July 26, 2023. doi:10.1001/jamanetworkopen.2023.25591

Data
Data available: Yes
Data types: Data (not involving human participants)
How to access data: We used publicly available data for this analysis. The dataset we constructed for this analysis is available upon request to Dr Varkila (mvarkila@stanford.edu).
When available: With publication

Supporting Documents
Document types: None

Additional Information
Who can access the data: anyone requesting the data
Types of analyses: validation
Mechanisms of data availability: with investigator support
